# Development and validation of derivative UV spectroscopic methods for simultaneous estimation of duloxetine and tadalafil in their binary mixtures: greenness-blueness evaluation

**DOI:** 10.1186/s13065-025-01483-5

**Published:** 2025-05-16

**Authors:** Dalia M. Nagy, Sayed M. Derayea, Al Amir S. Zaafan, Mohamed Oraby

**Affiliations:** 1 Analytical Chemistry Department, Faculty of Pharmacy, Minia University, Minia, 61519 Egypt; 2 Department of Pharmaceutical Analytical Chemistry, Faculty of Pharmacy, Sohag University, Sohag, 82524 Egypt

**Keywords:** Duloxetine, Tadalafil, Derivative spectra, Greenness evaluation

## Abstract

Antidepressant drugs, particularly selective serotonin reuptake inhibitors (SSRIs) and serotonin-norepinephrine reuptake inhibitors (SNRIs) are known to induce sexual dysfunction as a side effect. Duloxetine (DLX) and Tadalafil (TDL) are simultaneously determined in their pure state and laboratory-prepared mixtures by two novel, environmentally friendly, and accurate spectrophotometric methods. The first method based on second order derivatives while the second method is based on first derivative dual-wavelength detection. In method I, the linearity range was found to be 0.5–9 μg/mL and 1–14 μg/mL with limit of detection 0.15 μg/mL and 0.23 μg/mL for DLX and TDL, respectively, with a good correlation coefficient of 9998. In method II, the linearity range was found to be 1–10 μg/mL and 1–12 μg/mL with limit of detection 0.25 μg/mL and 0.20 μg/mL for DLX and TDL, respectively, with a good correlation coefficient of 0.9997 for DLX and 0.9998 for TDL. The validation of these methods followed the guidelines set by the International Council for Harmonization (ICH). The methods are accurate and precise. The proposed methods can be used for simultaneous determination of DLX and TDL in synthetic mixture. Additionally, the suggested method's greenness was assessed by means of four up-to-date ecological tools, namely the Eco-Scale, the National Environmental Method Index (NEMI), the Green Analytical Procedure Index (GAPI), and the Analytical Greenness metric approach and software (AGREE). The sustainability characteristics of the proposed method were also assessed using the Blue Applicability Grade Index (BAGI), a recently developed metric for assessing the practicality (blueness) of procedures.

**Supplementary Information:**

The online version contains supplementary material available at 10.1186/s13065-025-01483-5.

## Introduction

**Fig. 1:**
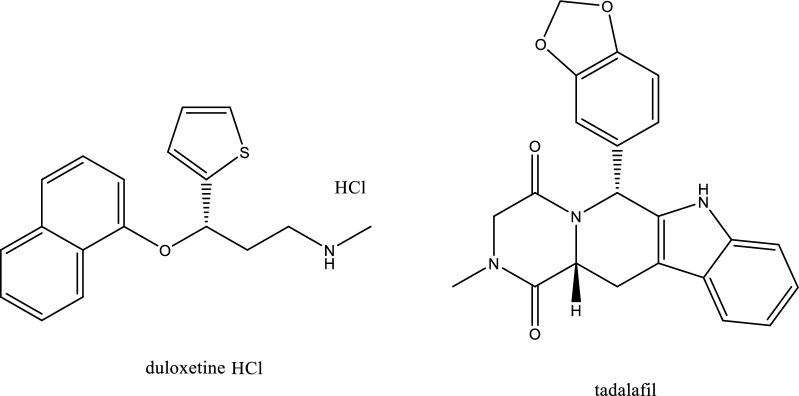
The chemical structures of the studied drugs

Duloxetine (DLX, Fig. 1), is a chemical compound with the name of 2( +)-(S)-Nmethyl-(gamma)-(1-naphthyloxy)-2 thiophen propylamine hydrochloride) [1]. It is plausible to infer that DLX functions as a selective reuptake inhibitor at serotonin (5HT) and norepinephrine (NE) carriers, primarily due to its low binding affinity for opioid, histaminergic, dopaminergic, glutamate, cholinergic, and gamma-aminobutyric acid (GABA) reuptake transporters [2]. DLX has been employed to treat depression and anxiety, offering benefits such as mood enhancement, improved sleep, reduced anxiety, and increased energy, and appetite [3]. Compared to other antidepressants, DLX has numerous benefits, including enhanced safety, increased efficacy, tolerance, and minimal undesirable effects. It also has dual inhibitory characteristics and a reduced affinity for neural receptors [4]. Literature concerning DLX encompasses various analytical methods such as spectrophotometry [2, 5–7], spectrofluorimetry [4, 8–10], TLC [1, 3, 11–13], electrochemical methods [14–17], gas chromatography [18], and HPLC [19–21]. Tadalafil (TDL, Fig. 1) is a chemical compound bearing the name of hydro-2-methyl-6-[3,4-(methylene dioxy)phenyl] pyrazino-[1’,2’:1,6] pyrido[3,4-b] indole-1,4-dione) [22]. TDL, a phosphodiesterase inhibitor, received approval in February 2003 and is utilized for managing erectile disfunction and impotence in men experiencing sexual function issues. It enhances sexual performance by augmenting blood flow in the veins of the penis [23] and assists in controlling pulmonary arterial hypertension and erectile dysfunction [24]. This inhibition increases cGMP levels in the smooth muscle of the penile arteries, leading to muscle relaxation, improved blood flow to the corpus cavernosum, and enhanced erectile function [25]. Several methods have been published for TDL analysis, including UV spectrophotometric [22, 26–28], spectrofluorimetric [23, 25, 29, 30], TLC [24, 31–33], HPLC [34–38], electrochemical [39–41] and capillary electrophoresis methods [42]. Spectrophotometric methods used for analysis of DLX suffered from certain limitations such as limited sensitivity [5, 6], or the use of harmful organic solvent [2], or damaging reagents [7]. While, the published spectrophotometric methods for TDL determination had drawbacks such as the involvement of extraction process using harmful organic solvent [22] or the use of concentrated acid which can be harmful and corrosive [27].

Antidepressant drugs, particularly selective serotonin reuptake inhibitors (SSRIs) and serotonin-norepinephrine reuptake inhibitors (SNRIs), such as duloxetine, are known to induce sexual dysfunction as a side effect [43]. The unpleasant sexual side effects including erectile dysfunction can be treated with TDL. Therefore, the focus of our investigation was the simultaneous determination of DLX and TDL. This research focuses exclusively on the simultaneous quantification of DLX and TDL. In compliance with the International Council for Harmonization (ICH) criteria, the current work presents a novel, environmentally friendly, accurate, and selective approach for determining DLX and TDL in bulk and laboratory-prepared mixtures. The innovation of the current work is due to that this is the first spectrophotometric method for simultaneous determination of DLX and TDL in their mixture. This study is entirely devoted to the development of a novel accurate and selective method for the determination of DLX and TDL in their mixture. Furthermore, the greenness of the proposed methods was confirmed by applying different green metric tools.

## Experimental

### Instrumentation

A T80 double beam UV–VIS spectrophotometer (PG instruments, Leicestershire, UK) and UV Win software were used to make the spectrophotometric measurements. The measurements were made using centimeter-sized quartz cells. Double-distilled Aquatron water still A4000D (Cole-Parmer, Staffordshire, UK) was used.

### Chemicals, standards, and samples

Mash Premiere Pharmaceuticals (Badr City, Cairo, Egypt) graciously supplied the DLX powder, and Andalous Pharma (6th of October city, Cairo, Egypt) provided the TDL powder. Cymbatex® 30 mg capsules (Eva Pharma, Giza, Egypt) and Tadalong® 20 mg tablets (Andalous Pharma, 6th of October City, Cairo, Egypt) were purchased from the local market. Methanol, HPLC grade solvent, was acquired from Merck in Darmstadt, Germany.

### Preparation of standard solution

A solution containing 1000 μg/mL of TDL and DLX was prepared by transferring 25 mg of each drug into 25-ml calibrated flasks and completed to the mark with methanol. Then, 0.5 mL of the TDL and DLX stock solution were taken into 10-mL volumetric flasks and diluted with the same solvent yielding 50 µg/mL standard solution. Then, Various concentrations of TDL and DLX were made using the same solvent. These solutions were put in the refrigerator for future use.

### Procedure for pharmaceutical preparations

From 10 tablets of Tadalong® 20 mg that have been finely powdered, a precisely weighed quantity of tablet powder equivalent to 50 mg of TDL was put into a 50 mL volumetric flask. The contents of ten capsules of Cymbatex® 30 mg were mixed well and precisely weighed. A quantity of the powder equivalent to 50 mg DLX was transferred into the same flask. Then, the medications are extracted with methanol by sonication for 30 min. Finally, the volume was up to 50 mL with the same solvent. The solution was filtered, and the first portion of the filtrate was discarded. Five determinations for each concentration were performed using the general assay procedure.

### Method development

#### Second derivative spectrophotometric method (Method I)

For employing the second order derivative method, portions of the DLX and TDL standard stock solutions were carefully transferred inside separate 10 mL volumetric flasks, and then the methanol was added. Every solution was subjected to scanning between 200 and 400 nm. Consequently, absorption spectra were derived, ranging from first to fourth order. For DLX and TDL analysis, second order derivative spectra were selected because of their superior linearity and sensitivity. After the calibration curves were generated, the concentration of each drug in the combination of drugs was estimated using the calibration curve data.

#### Dual-wavelength method of first derivative spectra (Method II)

The dual-wavelength method of the first derivative spectra was used to estimate DLX and TDL. This method necessitates two wavelengths for each drug. For one of the two drugs, the two wavelengths should have the same amplitude while the other drug has a different amplitude at each wavelength. DLX showed a noticeably different amplitude from TDL at the two selected wavelengths. The two other wavelengths were selected to ensure that the amplitude of TDL was considerably different from the amplitude of DLX which was similar.

## Result and discussion

**Fig. 2:**
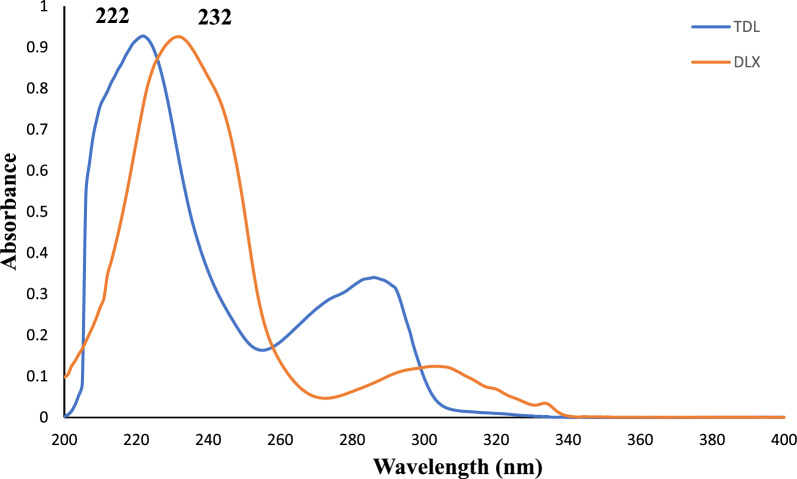
The zero-order UV absorption spectra of DLX and TDL (6 µg/mL) in methanol solvent

Concomitant administration of TDL with DLX provides effective treatment for sexual dysfunction resulting from the use of DLX. However, there is no reported method for the analysis of both drugs. Thus, there is for development of a new quantitative technique for the simultaneous assessment of the investigated medications. The present part introduces a new environmentally friendly spectrophotometric methods for the quantitation of DLX and TDL without physical separation. The proposed methods prove to be an efficient strategy for simultaneously analyze the two drugs, as it offers excellent selectivity, require minimal sample preparation and reduce solvent usage, making it a cost-effective and time-saving choice. The proposed approaches employ zero crossing point of the second derivative spectra dual wavelengths of the first derivative. Considering DLX and TDL substantial UV absorption and methanol solubility, methanol was subsequently chosen as the solvent for the current analytical techniques. As shown in Fig. 2, the zero-order UV spectra of DLX and TDL (6 μg/mL each) display overlay in their absorption bands. More than 90% of the spectral overlap was observed, highlighting how difficult it is to measure these medications by zero order UV spectra. Thus, mathematical approaches were utilized to get the derivative spectra of both drugs, beginning from the zero-order spectra and progressing up to four orders.

### Second derivative spectra (Method I)

**Fig. 3:**
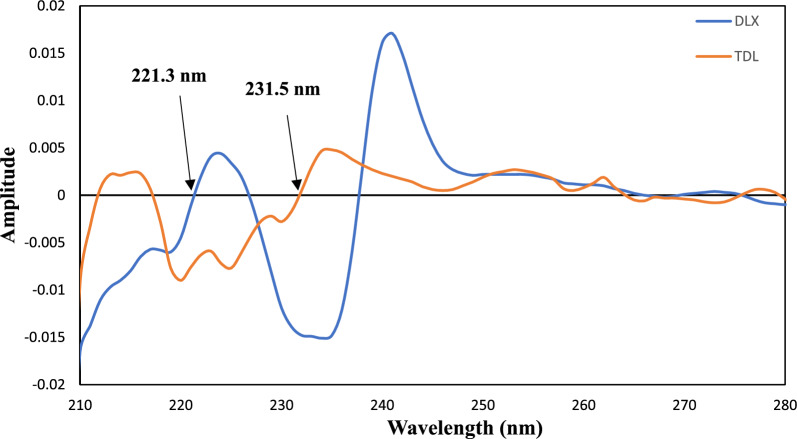
The second-order derivative UV spectra of DLX and TDL (6 µg/mL) in methanol solvent

The second-order derivative spectra of both drugs identified specific wavelengths that could be used for precise and linear detection of drugs. Because higher-order derivative spectra showed less linearity and sensitivity, only second-order derivative spectra were selected for quantitative analysis. Second derivative spectroscopy is performed using the TDL and DLX spectra that were obtained through scanning in methanol, depicted in Fig. 3. The D^2^ spectrum of DLX shows a zero-crossing point at 221.3 nm at which TDL could be determined easily. In the same way, the D^2^ spectrum of TDL shows a zero-crossing point at 231.5 nm at which DLX could be determined easily.

### Dual-wavelength method of first derivative spectra (Method II)

**Fig. 4:**
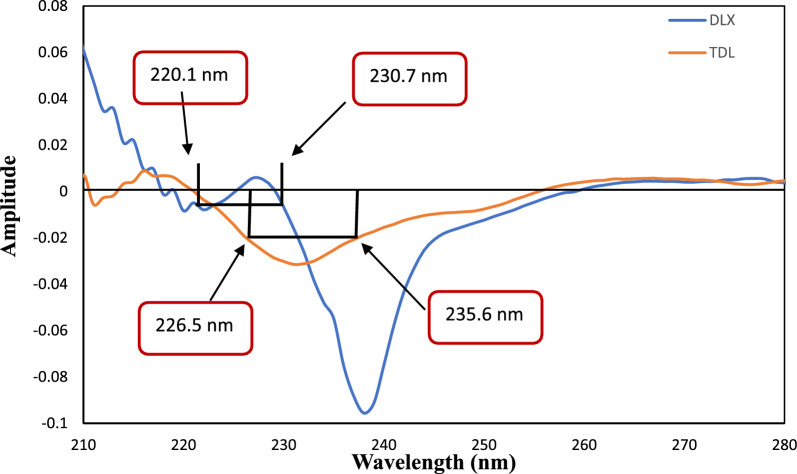
The first-order derivative UV spectra of DLX and TDL (6 µg/mL) in methanol solvent

The first derivative spectra of TDL and DLX at various concentrations demonstrated that TDL showed equal amplitude at 226.5 nm and 235.6 nm, on the other hand, DLX showed a significantly different amplitude. Similarly, different DLX concentrations displayed similar amplitude at 220.1 nm and 230.7 nm, while TDL demonstrated a notable variation in amplitude. Hence, the wavelengths 220.1 nm and 230.7 nm were chosen for the estimation of TDL without interference from DLX, while 226.5 nm and 235.6 nm were chosen for the estimation of DLX based on the aforementioned facts, Fig. 4.

### Method validation

The approaches were verified in terms of variables including, linearity range, accuracy, precision, LOQ, and LOD.

#### Linearity

In the second derivative method, the obtained calibration graphs for DLX and TDL were linear in the concentration range of 0.5–9 μg/mL and 1–14 μg/mL, respectively. The calibration curves employing the dual wavelength of the first derivative were linearly correlated to the drug concentrations throughout the range of 1–10 μg/mL for DLX and 1–12 μg/mL for TDL at 226.5, 235.6 nm and 220.1, 230.7 nm, respectively. The high values of correlation coefficients supported the excellent linearity of the calibration curves. The analytical data including the slope and intercept of the calibration curves has been summarized in tables.

#### LOQ and LOD

The LOD represents the minimum detectable amount of the analyte within a sample under the specified experimental conditions, although it is not measurable with precision. On the other hand, the LOQ signifies the smallest amount of the drug in a sample, which can be measured precisely and accurately. The LOD and LOQ values were calculated depending on the calibration graph's slope and intercept standard deviation. The ICH equations: LOD = 3.3SD/b and LOQ = 10SD/b (b = slope and SD = standard deviation of the intercept) were utilized. For the second derivative method, the LOD and LOQ values were found to be 0.13, 0.39 µg/mL for DLX, while the values were 0.23, 0.70 µg/mL for TDL. For the dual wavelengths of the first derivative method, the LOD and LOQ values were discovered to be 0.25, 0.76 µg/mL for DLX, while the values were 0.25, 0.75 µg/mL for TDL, indicating the adequate sensitivity of the proposed methods (Table 1).

**Table 1:** Statistical data of some analytical parameters of the proposed methods

Parameters	Method I		Method II	
	DLX	TDL	DLX	TDL
Linear range (µg/mL)	0.5–9	1–14	1–10	1–12
Slope ± SD	0.0024 ± 1.76E−05	0.0012 ± 1.01E−05	119.05 ± 0.0001	0.0053 ± 5.09E−05
Intercept ± SD	− 2.6E−05 ± 9.26E−05	0.0005 ± 8.42E−05	− 0.0015 ± 0.0009	− 0.0009 ± 0.0004
r *	0.9998	0.9998	0.9997	0.9998
*r*^2^ *	0.9996	0.9996	0.9994	0.9996
Number of determinations	5	5	5	5
LOD (µg/mL)	0.15	0.23	0.25	0.20
LOQ (µg/mL)	0.46	0.70	0.74	0.62

* r and r^2^ are correlation and determination coefficients

#### Accuracy

The average % recovery was calculated at five different levels of drug concentration (1, 3, 5, 7, and 9 µg/mL) for DLX and (2, 4, 6, 8, and 12 µg/mL) for TDL in triplicates for the two spectrophotometric methods, to assess the accuracy of the created approaches. The accepted % recovery (98.56–101.62), listed in Table 2, proved the excellent reliability of the proposed methods.

**Table 2:** Evaluation of the accuracy of the proposed methods for the determination of the investigated drugs

Drug	Conc. (µg/mL)	Method I		Method II	
		Amount found (µg/mL)	% Recovery ^a^ ± SD	Amount found (µg/mL)	% Recovery ^a^ ± SD
DLX	1	0.99	99.42 ± 1.40	0.99	99.78 ± 1.75
	3	2.96	98.57 ± 0.81	2.99	99.52 ± 1.89
	5	4.93	98.56 ± 1.69	5.05	100.97 ± 0.84
	7	7.04	100.64 ± 1.04	7.02	100.23 ± 1.59
	9	9.15	101.62 ± 1.36	8.93	99.20 ± 1.49
TDL	2	2.03	101.60 ± 1.91	1.99	99.48 ± 0.95
	4	3.95	98.84 ± 0.61	4.02	100.58 ± 1.86
	6	6.04	100.70 ± 1.47	5.99	99.84 ± 1.10
	8	8.09	101.10 ± 1.06	7.94	99.27 ± 1.87
	12	12.11	100.91 ± 0.93	12.13	101.08 ± 1.36

^a^Mean of three determinations

#### Precision

For the objective of repeatability and intermediate precision evaluation, the three-drug levels of concentration (1, 5, and 9 µg/mL) for DLX and (2, 6, and 12 µg/mL) for TDL were used in both methods. All measurements were performed in triplicates throughout a single day, and on three consecutive days in the repeatability and intermediate precisions, respectively. The %RSD was used to evaluate the precision of the constructed approaches. Table 3 lists the results of the suggested approaches, and their high precision was indicated by their small %RSD values (0.83–1.92).

**Table 3:** Evaluation of intra-day and inter-day precisions for the determination of the investigated drugs with the proposed methods

Method	Drug	Conc. (μg mL^−1^)	Intra-day precision		Inter-day precision	
			% Recovery ± SD^a^	%RSD	% Recovery ± SD^a^	%RSD
Method I	DLX	1	99.42 ± 1.40	1.41	99.64 ± 1.36	1.37
		5	98.56 ± 1.69	1.71	99.86 ± 1.69	1.69
		9	101.62 ± 1.36	1.34	100.47 ± 1.86	1.85
	TDL	2	101.60 ± 1.91	1.88	100.71 ± 1.93	1.92
		6	100.70 ± 1.47	1.46	99.53 ± 1.22	1.23
		12	100.91 ± 0.93	0.92	100.32 ± 1.34	1.34
Method II	DLX	1	99.78 ± 1.75	1.75	100.20 ± 1.44	1.44
		5	100.97 ± 0.84	0.83	100.52 ± 1.65	1.64
		9	99.20 ± 1.49	1.50	99.73 ± 1.19	1.19
	TDL	2	99.48 ± 0.95	0.95	100.11 ± 1.75	1.75
		6	99.84 ± 1.10	1.10	100.68 ± 1.41	1.40
		12	101.08 ± 1.36	1.34	100.82 ± 0.97	0.96

^a^Mean of three determinations

### Application to pharmaceutical dosage forms

Using the previously mentioned procedures, it was possible to successfully determine DLX and TDL in the pharmaceutical dosage forms. A statistical analysis was conducted to compare the outcomes of the proposed approaches with those attained by reported technique [33]. To compare the two approaches statistically, the t-test and F-test were used. The F- and t-test calculated values were less than their 95% confidence level reference values, indicating that there were no discernible variations in accuracy and precision between the recommended and published methods (Table 4).

**Table 4:** Comparison between the results of the estimation of DLX and TDL in their pharmaceutical preparations using the proposed and reported methods

Parameter	Method I		Method II		Reported method	
	DLX	TDL	DLX	TDL	DLX	TDL
Mean % recovery^a^	99.53	100.09	100.31	99.76	100.42	101.32
SD	1.20	1.53	1.26	1.25	0.65	1.63
t-test^b^	1.46	1.23	0.17	1.70		
F-value^b^	3.41	1.13	3.76	1.70		

^a^Mean of five measurements

^b^Tabulated value at 95% confidence limit, F = 6.388 and t = 2.306

### Greenness assessment

The green analysis is described as having no or minimal usage of dangerous chemicals, the elimination of waste, and a decrease in energy consumption. To evaluate the degree of greenness of the analytical procedure, it is highly recommended to utilize certain advanced tools [44–49]. The technique related to the NEMI was utilized to evaluate the greenness of the proposed methods [50]. It depends on the use of non PBT. Methanol used in the developed spectrophotometric methods is not a PBT solvent. Furthermore, the waste volume was not greater than 50 mg or 50 mL. According to these results, the proposed methods conserved solvents while producing low amounts of waste. As a result of these factors, the proposed methods achieved each of the four quadrants of the greenness profile and is regarded as a green approach, as shown in Fig. 4.

The Eco-scale is a straightforward method used for the assessment of the analytical method greenness [51]. The subsequent equation (analytical Eco-Scale score = 100 – total penalty) is used to calculate total score. A penalty point was assigned for each procedure's defined parameters, such as the number of chemicals used, dangers to employees, waste products, and consumption. After that, the total penalty was calculated by summation of these penalty points. If the score exceeds 75, the analytical method is considered green. The Eco-scale score of the developed spectrophotometric methods was found to be 91 (Table 5), thus, it is regarded as environmentally friendly.

**Table 5:** Evaluation of the greenness of the proposed methods using the Eco scale score method

Parameters		Penalty points
Reagents	Methanol	6
Heating	–	0
Temperature	25 °C	0
Cooling	–	0
Technique	Spectrophotometry	
Energy (kWh per sample)	(< 0.1)	0
Occupational hazard	(Analytical process hermitization)	0
Waste	(10 mL)	3
Total penalty points		9
Analytical eco-scale total score		91

^a^If the score is greater than 75, it represents excellent green analysis. If the score is greater than 50, it represents an acceptable green analysis. If the score is less than 50, it represents inadequate green analysis

Another trend, called GAPI, can be used to assess how environmentally friendly an analytical process is, from collecting samples to final analysis. The greenness of each stage in the analysis process is evaluated and the result was expressed as a pictogram, which has three color levels, green, yellow, and red [52]. The suggested technique revealed 4 yellow, 8 green, and 3 red areas when evaluated using the GAPI metric.

The most recent metric is AGREE. The submitted criteria for the AGREE metric are flexible and can be weighted differently, drawing design ideas from the 12 significance principles of the green chemistry. Twelve input variables are all graded from 0 to 1 [53]. The result is a graph that resembles a clock, with the overall score and a color representation in the middle. The evaluation can be done with free software, which generates a report and an auto-generated graph. The AGREE evaluation reveals that the suggested method's score is 0.75 depends on numerous variables, including solvent's type and its volume used, and solvent toxicity to individuals and the surrounding environment. As a result, the suggested strategy has a minimal environmental impact (Fig. 4).

### Blueness evaluation

**Fig. 5:**
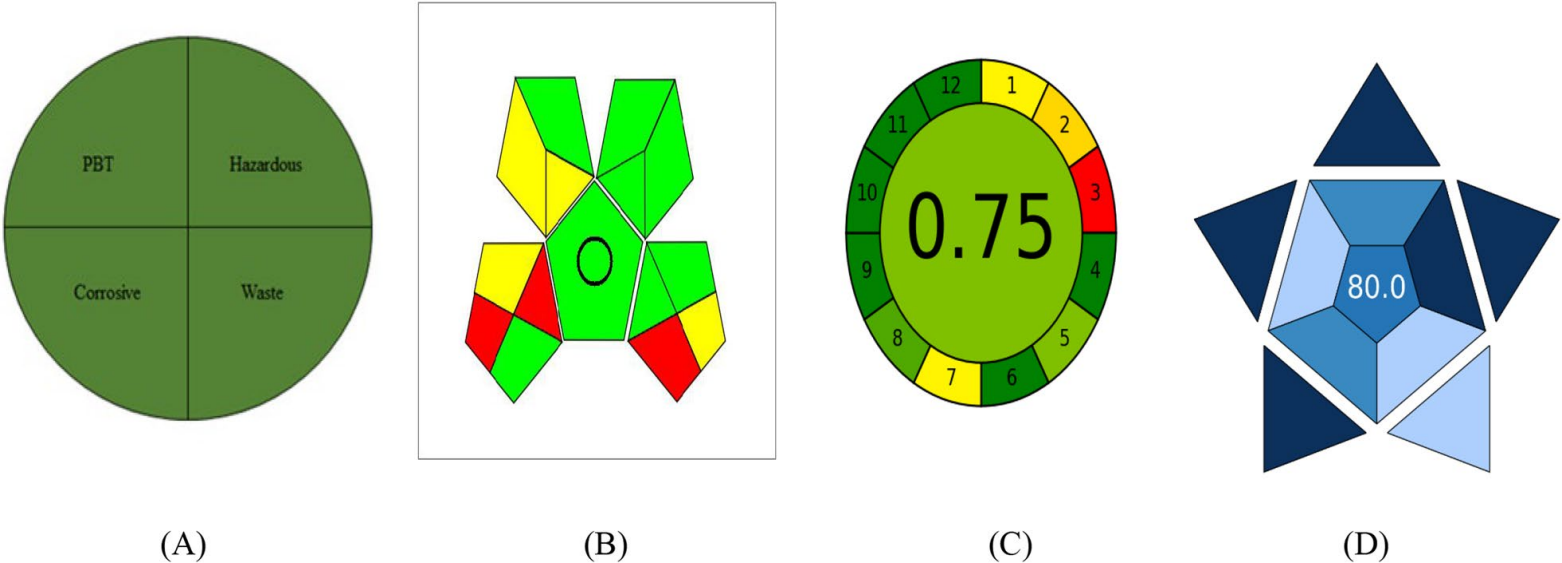
Evaluation of the proposed spectrophotometric methods using NEMI pictograms (**A**), GAPI (**B**), AGREE (**C**), and BAGI (**D**) tools for greenness and blueness

A new metric tool, the Blue Applicability Grade Index (BAGI), is presented to assess the analytical method's practical considerations [54]. The BAGI metric tool yields two sets of results: an asteroid-shaped pictogram as a graphical illustration and a score in numbers at the center of the pictogram. The asteroid-shaped pictogram, comprised of several shades of blue color representing varying degrees of compliance (dark blue for high, blue for moderate, light blue for low, and white for non-compliance), visually depicts the assessment result. To generate a pictogram and a score that illustrate the usefulness and effectiveness of an analytical approach, BAGI considers 10 factors (Table S1). It is advised that the final score be greater than 60 for the analytical procedure to be practical. The suggested approach receives an overall score of 80 in the final rating shown in the pictogram's center (Fig. 5).

## Conclusion

New green spectrophotometric methods were developed to measure pharmaceutical forms of DLX and TDL as well as laboratory-prepared mixtures. The first method is based on second order derivative spectra while the second method is based on dual-wavelength of first derivative spectra. The methods are novel, environmentally friendly, and accurate. For green spectrophotometric determination of the studied drugs simultaneously, the procedures' level of greenness was evaluated using a number of tools namely, NEMI, AGREE, GAPI, and the eco-scale. The obtained results demonstrated that the proposed methods have a high greenness level and confirmed that neither the environment nor the health of the analysts were impacted by the developed models. The proposed methods can be used for regular quality control tests for directly determining DLX and TDL either separately or concurrently in a binary combination with no prior separation. The procedure was analytically evaluated according to the International Council of Harmonization.

## Supplementary Information

Additional file 1.

## Data Availability

The datasets used and/or analyzed during the current study are available from the corresponding author on reasonable request.
