# Supplementary material for: Development and validation of derivative UV spectroscopic methods for simultaneous estimation of duloxetine and tadalafil in their binary mixtures: greenness-blueness evaluation
Source: BMC Chem. 2025 May 16;19(1):130. doi: 10.1186/s13065-025-01483-5 (PMC12083154; doi:10.1186/s13065-025-01483-5)
Supplement: Supplementary file 1 — Additional file 1. [file 13065_2025_1483_MOESM1_ESM.docx]

**S1 (Table).** The 10 factors utilized in evaluation of the proposed method using Blue Applicability Grade Index (BAGI)

| **Parameter** | **Rating** | **Remarks** |
| --- | --- | --- |
| 1. Type of Analysis | moderately blue | Method is categorized as quantitative |
| 1. Multi-Analyte Procedure | light blue | It determines two components |
| 1. Analytical Technique Used | moderate blue | A spectrofluorometer device was used, which is easily accessible in most labs |
| 1. Simultaneous Sample Preparation | light blue | The suggested method's simultaneous preparation's ease of use and time-saving nature |
| 1. Sample Preparation | moderately blue | It involves little and no payment for sample preparation |
| 1. Samples Per Hour | dark blue | Large number of samples |
| 1. Availability of Reagents | dark blue | there are no derivative reagents—common reagents that are sold commercially |
| 1. Preconcentration | dark blue | it doesn't require preconcentration |
| 1. Automation of Device | white | Procedures with manual devices |
| 1. Amount of Sample | moderate blue | The sample volume is small and direct fluorometric technique |

**S2 (Table).** Comparison of the greenness and blueness between the proposed and reported methods.

|  | **Proposed method** | **Reported method** [1] |
| --- | --- | --- |
| GAPI | *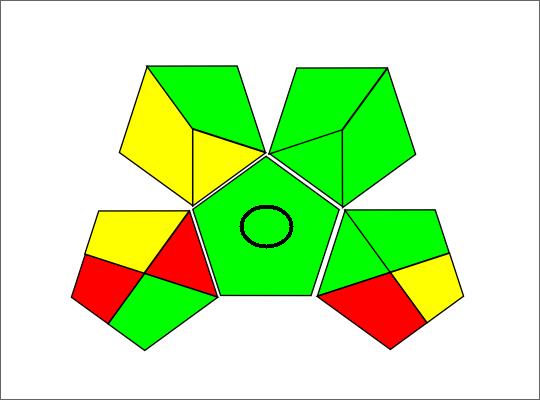* | 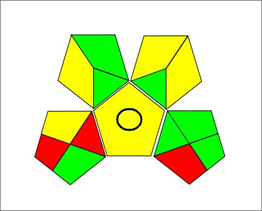 |
| AGREE | *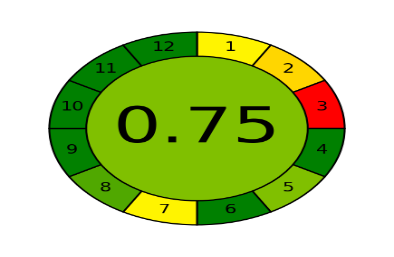* | 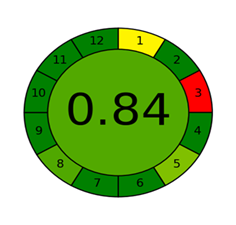 |
| BAGI | *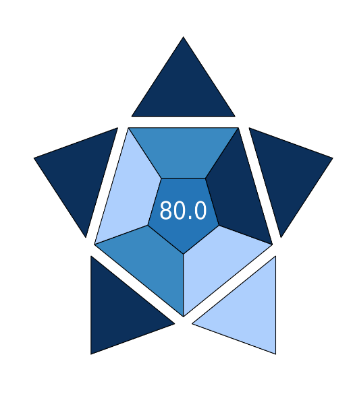* | 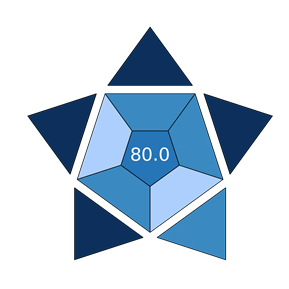 |
